# The rates of stem cell division determine the cell cycle lengths of its lineage

**DOI:** 10.1016/j.isci.2021.103232

**Published:** 2021-10-06

**Authors:** Purna Gadre, Nitin Nitsure, Debasmita Mazumdar, Samir Gupta, Krishanu Ray

**Affiliations:** 1 Department of Biological Sciences, Tata Institute of Fundamental Research, Mumbai, Maharashtra 400005, India; 2 School of Mathematics, Tata Institute of Fundamental Research, Mumbai, Maharashtra 400005, India; 3 Biology Department, Indian Institute of Science Education and Research, Pune, Maharashtra 411008, India; 4 Department of Molecular and Life Sciences, University Zurich, 80006 Zürich, Switzerland

**Keywords:** Cell biology, Stem cells research, Bioinformatics

## Abstract

Adult stem cells and their transit-amplifying progeny alter their proliferation rates to maintain tissue homeostasis. To test how the division rates of stem cells and transit-amplifying progeny affect tissue growth and differentiation, we developed a computation strategy that estimates the average cell-cycle lengths (lifespans) of germline stem cells and their progeny from fixed-tissue demography in the *Drosophila* testis. Analysis of the wild-type data using this method indicated that during the germline transit-amplification, the cellular lifespans extend by nearly 1.3-fold after the first division and shrink by about 2-folds after the second division. Cell-autonomous perturbations of the stem cell lifespan accordingly altered the lifespans of successive transit-amplifying stages. Remarkably, almost 2-fold alterations in the lifespans of stem cells and their immediate daughters did not affect the subsequent differentiation. The results indicate that the early germline division rates can adjust the following division rates and the onset of differentiation.

## Highlights

• Prediction of cellular lifespan from the demography of transit-amplifying cells
• Lifespans of spermatogonial cells change anomalously during transit-amplification
• Anomalous lifespan extension during transit-amplification precedes the onset of Bam
• Lifespan changes of early TA stages readjust that of the subsequent stages

## Graphical abstract

**Figure:**
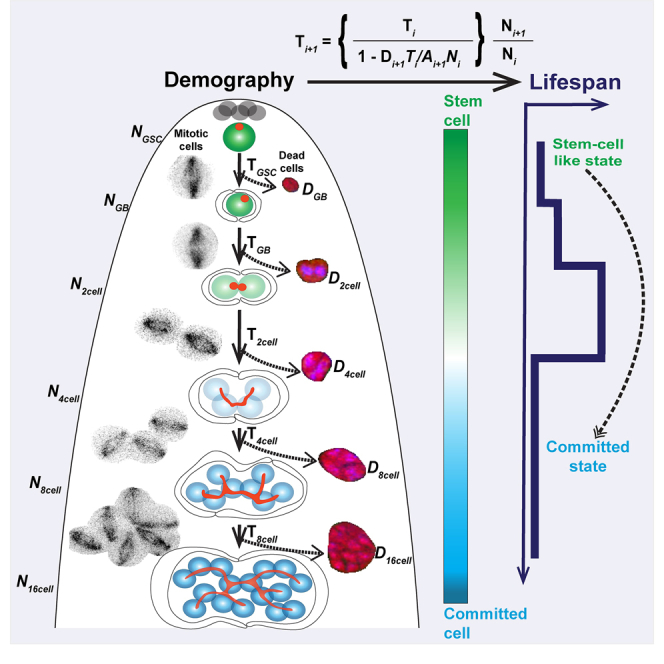

## Introduction

Many adult stem cells produce progenitors, which undergo transit-amplifying (TA) divisions before terminal differentiation. Hormonal stimulation (Di Gregorio et al., 2001; Giraddi et al., 2015), aging (Charruyer et al., 2009; Paliouras et al., 2012; Tomasetti et al., 2019), tissue damage (Lehrer et al., 1998; Ichijo et al., 2017), etc., are indicated to alter the division rates of stem cells and their progeny. Previous studies have shown that the TA cells pass through a continuum of transcriptomic states, which sets the time of differentiation – independent of the TA cell cycle rates (Gao et al., 1997; Insco et al., 2009; Cinquin et al., 2010). This conjecture appears inconsistent because coordination of this autonomous differentiation clock with rates of TA divisions is essential for tissue homeostasis, and defects in this process can lead to cancer or other disorders (Clarke and Fuller, 2006; Li and Laterra, 2012; Janssens and Lee, 2014; Zhang and Hsu, 2017). Despite its importance, it is unclear whether the rates of stem cell and TA divisions influence the differentiation clock.

*Drosophila* spermatogenesis provides an ideal model system to study the regulation of TA divisions. Accumulation of a translational repressor, Bag-of-marbles (Bam), to an optimum level arrests the TA divisions after the fourth round, suggesting that the bam expression and degradation kinetics could set the differentiation clock (Insco et al., 2009). It was also evident that slowing down the third and fourth TA divisions (of the 4- and 8-cell stages, respectively) could induce premature germline differentiation after the third round. However, the effect was not fully penetrant (Insco et al., 2009) as several cysts were shown to conclude the TA and meiosis in a wild-type-like manner in the cell cycle perturbed backgrounds. This observation also suggests that the differentiation clock can adjust to accommodate changes in the rates of TA divisions. Further, perturbation of the GSC and early TA divisions did not affect the differentiation at the 16-cell stage (Gadre et al., 2020). These investigations highlight that the rates of TA divisions could influence the differentiation clock up to a limited extent. However, we still lack clarity regarding the quantitative limit of this readjustment.

To resolve this issue, one requires an estimate of how the cell cycle lengths of GSCs and TA cells change under different conditions. Previous studies inferred the changes in the proliferation rates of the GSCs and TA cells by enumerating phospho-histone3/BrdU stained clusters (Li et al., 2007; Parrott et al., 2012; Gupta et al., 2018; Gadre et al., 2020) and performing BrdU/EdU pulse-chase analysis (Insco et al., 2009; Monk et al., 2010). Although these methods presented a comparative measure to examine how different factors regulate the GSC and TA pool, they failed to quantitate the cell cycle lengths of the GSC and TA stages. The time between two successive GSC divisions (inter-division lifespan) was recently estimated using time-lapse imaging of isolated testes for up to 19 h (Sheng and Matunis, 2011; Lenhart and DiNardo, 2015). Although time-lapse imaging measures the exact length of the cell cycle, it is tedious for assessing the effects of a multi-factor manipulation of the cell cycle rates. Moreover, long-term exposure of cells to light while imaging increases phototoxicity because of ROS generation, which might alter the very rate it is supposed to measure.

Therefore, we devised an optimized computation strategy to predict average cell cycle lengths, hereafter referred to as the ‘lifespans’, of the GSCs and individual TA stages using five independently estimated parameters: (1) Sizes of GSC and stage-wise TA population, (2) GSC mitotic indices, (3) GSC M-phase duration, (4) Germ-cell death frequency, and (5) Persistence time of a dead cyst. This method restricted the requirement for time-lapse imaging and enabled us to examine the effects of a range of genetic perturbations on the GSC and TA division rates. Using this method, we probed the correlation between the GSC division rates and the onset of the differentiation program. The lifespan predictions obtained from the experimental data using this method indicate that altering the rates of stem cell divisions and early TA divisions could affect the cell cycle lengths during subsequent divisions. In addition, it could potentially readjust the differentiation clock to such an extent that the scale and relative pattern of the germline amplification would remain unaltered.

## Results

### Formulation of the equation for estimation of the cellular lifespans of the TA stages

**Figure 1:**
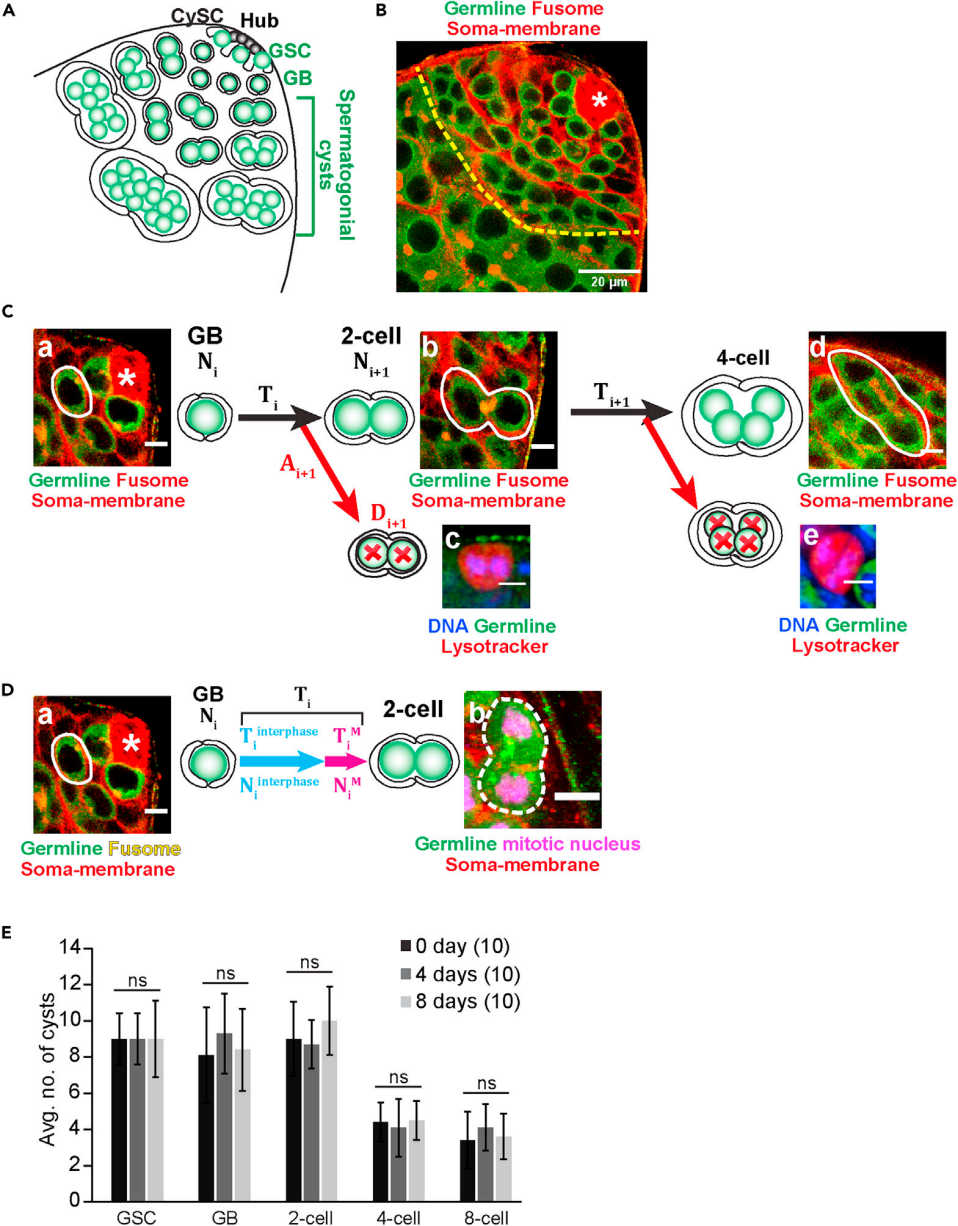
Formulation of the equation for empirical estimation of the cell life spans of the TA stages (A) Schematic illustrates the process of transit-amplification during early spermatogenesis. Glossary: GSC – Germline Stem Cell, CySC – Cyst Stem cell, and GB – Gonialblast. (B) The apical tip of wild-type (*CantonS*) testis stained for Vasa (Germline, Green), Armadillo (Somatic membrane, Red), and Adducin/Hts (Fusome, Red). (Scale bars ∼20 μm). (C) Schematic describes the sequential method (Equation (7)) of time estimation. $T_{i}$ and $T_{i+1}$ denote the time taken for a GB to 2-cell cyst transition (GB life span) and a 2-cell to 4-cell transition (2-cell life span), respectively. $N_{i}$ and $N_{i+1}\;$denote the number of GBs (A) and 2-cell cysts (B), respectively. $D_{i+1}\;$ denotes the number of 2-cell dead cysts, depicted by the Lysotracker (Red)-positive 2-cell cyst (C). $A_{i+1}$ denotes the persistence time of a dead 2-cell cyst. (Scale bars ∼5 μm). (D) Schematic describes the individual method (Equation (2)) of time estimation. $N_{i}^{interphase}$ and $N_{i}^{M}$denote the number of GBs in G1/S/G2 phases (A) and M-phase, marked by phospho-Histone3-positive (pH3, mitotic nucleus, Magenta) GB (B), respectively. $T_{i}^{interphase}$ and $T_{i}^{M}$denote the duration of G1+S + G2 phases and that of the M-phase of GBs, respectively. $T_{i}$ denotes the GB life span. (Scale bars ∼ 5 μm). (E) Histograms show the relative stage-specific distribution profile of cysts (average ± SD) in *CantonS* adults aged 0-, 4- and 8-days after emergence from the pupal case (eclosion) at 29°C. (n = 10 for each group. Kruskal–Wallis ANOVA test, p > 0.05 for all stages).

The GSCs surround the stem cell niche in wild-type testes, termed as the hub (Figures 1A and 1B). Each TA division displaces the resultant cyst further away from the hub. The GSCs and the TA stages can be visualized by immunostaining the testes for Vasa (labels the germline cells), Armadillo (labels the hub and the cyst perimeter), and MAb1b1/Hts1 (labels spectrosomes and fusomes; Figure 1B). To compute the lifespans of the GSCs and TA cells, we assumed the following:

1) The TA population in the adult testis is in a steady state (Figure 1E).
2) The lifespan of each stage (GSC and TA stages) remains invariable.
3) The cell cycle phases at each stage (GSC and TA stages) are uniformly distributed across the cells in that stage.

In such a closed system, at steady-state, the relative population of a stage $\left(N_{i}/\sum_{i=1}^{i=4}N_{i}\right)$ is equal to its relative lifespan$\left(T_{i}/\sum_{i=1}^{i=4}T_{i}\right)$) if no germline cysts are lost due to death. Here, i represents the TA stage (taking four values corresponding to the four TA stages: GB, 2-cell, 4-cell, 8-cell), $N_{i}$ represents the average number of cysts at stage i, and $T_{i}$ denotes the lifespan of the stage i, respectively (see STAR Methods). For two successive stages i and i+1, this relationship can be expressed as,

(Equation 1) $$\frac{N_{i}}{N_{i+1}}=\frac{T_{i}}{T_{i+1}}\;$$

### Adaptation of the computation strategy to account for the germ-cell death

Reportedly, a small number of cysts undergo germ-cell death (GCD) at every TA stage (Yacobi-Sharon et al., 2013; Yang and Yamashita, 2015; Chiang et al., 2017). Hence, only a fraction of cysts successfully transitions to the next stage. In our model, we assumed that, for a transition of a cyst from stage i to i+1, the GCD occurs after stage i cysts complete the cell cycle (i.e., concluding the G1, S, G2, and M), and before they enter the next cell cycle of stage i+1. To account for this loss, we defined the survival probability (*s*_*i*+1_) as the possibility of a successful transition from stage i to i+1 (see STAR Methods). Incorporating this survival probability (*s*_*i*+1_) in Equation (1) gives,

(Equation 2) $$\;\frac{N_{i+1}}{N_{i}}=\frac{s_{i+1}*\;T_{i+1}}{T_{i}}\;$$

$s_{i+1}$ can be calculated by using the same logic as Equation (2): the fraction of dead spermatogonial cysts $\left(D_{i+1}/N_{i}\right)$ during the transition from stages i to i+1 is equal to the product of the probability of GCD $\left(d_{i+1}=1-s_{i+1}\right)$ and the relative persistence time of the dying cysts $\left(A_{i+1}/T_{i}\right)$. $D_{i+1}\;$denotes the average number of cysts that died during the transition from stage i to stage i+1, and $A_{i+1}$ denotes the time for which a dead cyst remains visible in the testis (persistence time). This relationship is expressed as:

(Equation 3) $$\frac{D_{i+1}}{N_{i}}=\frac{\left(1-s_{i+1}\right)\;A_{i+1}}{T_{i}}$$

After a rearrangement of Equation (3) we get,

(Equation 4) $$s_{i+1}=1-\;\frac{D_{i+1}T_{i}}{A_{i+1}N_{i}}$$

### Solving the equations to obtain the lifespans

1. Sequential estimation method (Figure 1C)

Equations (2) and (4) give two equations in the three unknowns $T_{i\;},\;T_{i+1}$ and $s_{i+1}$*.*

(Equation 5) $$\frac{N_{i+1}/N_{i}}{T_{i+1}/T_{i}}=s_{i+1}=1-\frac{D_{i+1}T_{i}}{A_{i+1}N_{i}}$$

### Eliminating $s_{i+1}$ and solving for $T_{i+1}$ gives

(Equation 6) $$T_{i+1}=\frac{N_{i+1}}{N_{i}}\left(\frac{T_{i}\;}{\;1-D_{i+1}T_{i}/A_{i+1}N_{i}}\right)$$

2. Individual estimation method (Figure 1D)

Alternatively, the cyst population of stage i can be divided into two sub-stages: the cyst population in G1/S/G2 phases and M phase. We denote these two stages as stage$\;i^{interphase}$, and$\;i^{M}$, respectively. As mentioned earlier, our assumption implies that no deaths occur during the phases G1, S, G2, and M of the germline cell cycle. As the sub-stage $i^{interphase}$ is by definition made up of the phases G1, S, and G2, and as the sub-stage $i^{M}$ is the corresponding M-phase, the assumption implies that any spermatogonial cyst in sub-stage $i^{interphase}$ has a 100% probability of transitioning to the sub-stage $i^{M}$. Consequently, we have,

(Equation 7) $$\frac{N_{i}^{M}}{N_{i}}=\frac{T_{i}^{M}}{T_{i}}\;$$

where $T_{i}^{M}$ and $T_{i}$ are M-phase length and the lifespan of the stage i, respectively. $N_{i}^{M}$ and $N_{i}$ are the number of cysts of stage i in M-phase and the total number of cysts of stage i, respectively. The above relationship shows that the fraction of cysts of stage i in M-phase (Mitotic index$\;N_{i}^{M}/N_{i}$) is equal to the length of the M-phase relative to the total lifespan of stage *i* $\left(T_{i}^{M}/T_{i}\right)$.

### The TA population in the adult testis is in a steady-state

To explore the germline population dynamics in the adult testis, we enumerated the GSCs and TA stage-wise distribution of the cysts from (1) freshly emerged (0-day), (2) 4-day old, and (3) 8-day old wild-type males. The cyst distribution remained invariant, demonstrating that the germline TA population is in a steady state until 8-days post eclosion (Figure 1E).

### GSCs and TA cysts take nearly equal time to complete their M-phases

**Figure 2:**
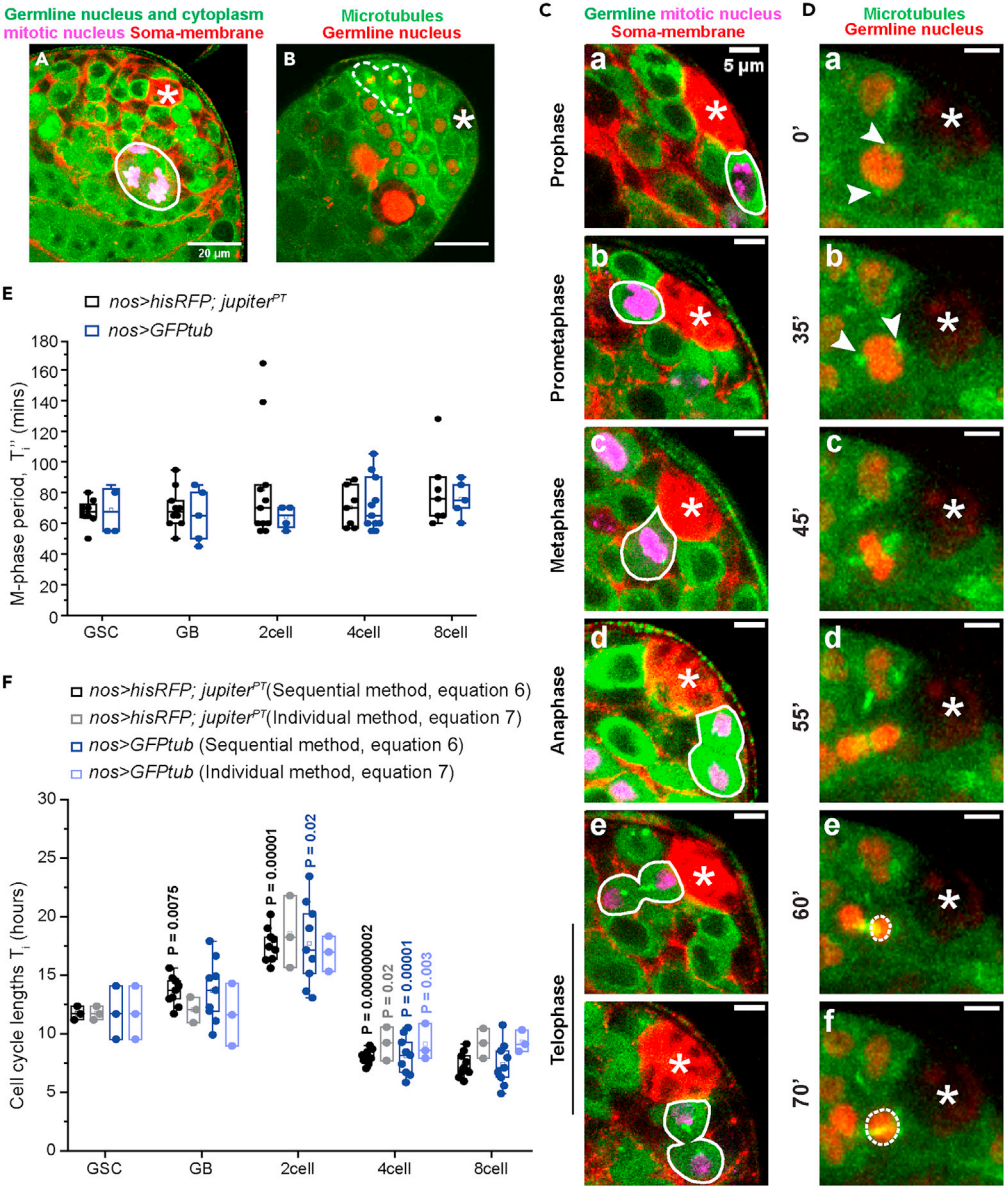
Time-lapse recording of the GSCs M-phases (A) Apical tip of *nos > hisRFP* (Germline nucleus, Green) testis stained for Vasa (Germline cytoplasm, Green), Armadillo (Somatic membrane, Red), and pH3 (Mitotic nucleus, Magenta). The white boundary marks a pH3-positive 4-cell cyst (Scale bars ∼20 μm). (B) A snapshot from the time-lapse imaging of the apical tip of *Jup*^*PT*^ (Microtubules, Green); *nos > hisRFP* (Germline nucleus, Red) testis. Dotted white boundaries mark a 4-cell cyst in metaphase. (Scale bars ∼20 μm). (C) Adult testis tip stained for Vasa (Germline, Green), Armadillo (Soma membrane, Red), and pH3 (Mitotic nucleus, Magenta), showing a GSC in prophase (A), pro-metaphase (B), metaphase (C), anaphase (D) and telophase (E and F) marked by white boundaries (Scale bars ∼5 μm). (D) Montage of a time-lapse image of a GSC (*Jup*^*PT*^(Green); *nos > hisRFP*(Red)) undergoing prophase (A), prometaphase (B), metaphase (C), anaphase (D) and telophase (E and F). Arrowheads mark the position of the separated centrosomes. White dashed circles show the increase in nuclear size. Time intervals in minutes are indicated next to the image panels. (Scale bars ∼ 5 μm). In all images, the asterisk marks the hub. (E) Box plots show the duration of the M-phase in GSCs and TA stages in control backgrounds, namely, *nos > hisRFP; jup*^*PT*^ (number of time lapse images analyzed = 26) and *nos > GFPtub* (number of time lapse images analyzed = 21) (p values calculated using Kruskal Wallis ANOVA). (F) Box plot shows the life spans of GSCs and TA stages in control backgrounds, namely, *nos > hisRFP; jup*^*PT*^ and *nos > GFPtub,* using Equations (6) and (7). (p values calculated using Student’s T test). See also Figures S2 and S3.

Phospho-Histone-3 (pH3) staining in fixed preparation efficiently identified the mitotic stages from prophase to telophase (Figures 2A and 2B (Giet and Glover, 2001)). To determine the M-phase length of the GSCs and cysts in live preparations, we collected time-lapse images from *nosGal4vp16>UAS-histone-RFP; jupiter-GFP* (*nos > hisRFP; jup*^*PT*^) testes *ex vivo* (Videos S1, S2, S3, S4, and S5). *nos > hisRFP* expression labeled the nuclei of GSC and its progeny cells (Figures 2A, 2B, and 2D). The M-phase was discerned by the microtubule spindle morphology marked by Jupiter-GFP as per the established criterion (Siller et al., 2005; Karpova et al., 2006). The appearance of two centrosome-associated microtubule clusters was considered the beginning of prophase (arrowhead, Figures 2D–2A (Giet and Glover, 2001; Savoian and Rieder, 2002)). Metaphase was identified by the characteristic spindle formation and chromatin alignment at the cell equator (arrow, Figures 2D–2C). The spindle was then resolved through anaphase (Figures 2D–2D) and telophase (Figures 2D and 2E). A visible increase in the nuclear size after anaphase was considered as the end of telophase (Figures 2D–2F). The GSC M-phase period varied from 50 to 80 min (Figure 2E). We used the median of this dataset (67.5) for our calculations. The M-phase durations remained invariant throughout the transit-amplification (Figures S2 and 2E).

Video S1. A GSC undergoing mitosis marked by noGal4vp16>UAS-hisRFP (Red) and microtubules marked by JupPT (Green), related to Figure 2 Asterisk denotes the hub. Arrowheads indicate the separated centrosomes. Yellow circles mark the increase in nuclear size at the end of telophase. (Scale Bars ∼10μm).

Video S2. A GB undergoing mitosis marked by noGal4vp16>UAS-hisRFP (Red) and microtubules marked by JupPT (Green), related to Figure 2 Asterisk denotes the hub. Arrowheads indicate the separated centrosomes. Yellow circles mark the increase in nuclear size at the end of telophase. (Scale Bars ∼10μm).

Video S3. 2-cell spermatogonia undergoing mitosis marked by noGal4vp16>UAS-hisRFP (Red) and microtubules marked by JupPT (Green), related to Figure 2 Asterisk denotes the hub. Arrowheads indicate the separated centrosomes. Yellow circles mark the increase in nuclear size at the end of telophase. (Scale Bars ∼10μm).

Video S4. 4-cell spermatogonia undergoing mitosis marked by noGal4vp16>UAS-hisRFP (Red) and microtubules marked by JupPT (Green), related to Figure 2 Asterisk denotes the hub. Arrowheads indicate the separated centrosomes. Yellow circles mark the increase in nuclear size at the end of telophase. (Scale Bars ∼10μm).

Video S5. 8-cell spermatogonia undergoing mitosis marked by noGal4vp16>UAS-hisRFP (Red) and microtubules marked by JupPT (Green), related to Figure 2 Asterisk denotes the hub. Arrowheads indicate the separated centrosomes. Yellow circles mark the increase in nuclear size at the end of telophase. (Scale Bars ∼10μm).

We also expressed *UAS-GFP-tubulin84B* (*GFP-tub*) transgene using *nosGal4vp16* to mark the germline spindles (Figure S3). Time-lapse imaging revealed that the M-phase durations remain the same in the *nos > GFP-tub* background as compared to *nos > hisRFP; jup*^*PT*^ (Kruskal-Wallis ANOVA, Figure 2E).

### The GSCs divide every 12 hours

Consistent with the previous report (Hasan et al., 2015), no GCD was recorded in GSCs in wild-type testis (N = 54). Therefore, we calculated the average time between two successive GSC divisions (inter-division lifespan) directly from the M-phase period (Figure 2E) and GSC mitotic index (Table S1) using Equation (2). The average GSC life span in control background (*nos > eGFP)* was estimated to be 11.7 ± 0.6 h using *nos > hisRFP; jup*^*PT*^ data and 11.8 ± 2.3 h using *nos > GFP-tub* data (Figure 2F), which falls within the reported range (Lenhart and DiNardo, 2015).

### Cellular life spans increase for the first two TA divisions and then contract by nearly 2-folds

To estimate the life spans of the TA stages using Equation (1), we utilized the consolidated, stage-wise cyst counts ($N_{i}$ and *N*_*i*+1_’s; Table S2) obtained from the previously published literature (Gadre et al., 2020). The number of Lysotracker-positive cysts in Phase-I from the same genetic background was considered as the *D*_*i*+1_’s (Table S3). The stage-wise Phase-I persistence time (*A*_*i*+1_’s) obtained from the time-lapse images (Figure S1A; Table S4) varied substantially across samples, ranging from one to four hours (Table S4). A limited simulation suggested that for *A*_*i*+1_ values between 1.5 and 5 h, the life span estimates (*T*_*i*_’s) remain fairly unaltered (Figure S1C). This outcome justified our assumption of the average of the observed values of Phase-I persistence time as a constant value for *A* (Table S4).

The analysis revealed the life span of GBs (13.6 ± 1.2 h) is about 16% longer than that of the GSCs. The 2-cell life spans (17.6 ± 1.6 h) were prolonged further by approximately 29% (Figure 2F). Subsequently, the 4-cell life spans contracted by about 54% (8.1 ± 0.6 h), and the 8-cell life spans remained at a similar level (7.3 ± 1.1 h; Figure 2F). These observations were consistent with a previous report suggesting that the 4- and 8-cell cysts take more than 7 h to complete their cell cycle (Insco et al., 2009).

To confirm these estimates, we sought to calculate the life spans by an alternate method using Equation (2), which utilizes the stage-wise mitotic indices (Table S1) and the stage-wise M-phase durations (Figure 2E; Table S5). Consistent with the results obtained using Equation (1), these estimates indicated similar life span changes at the 2- and 4-cell stages (Figure 2F). Furthermore, life span calculations using the M-phase durations obtained from time-lapse imaging of *nos > GFP-tub* testes also yielded similar results (Kruskal–Wallis ANOVA, Figure 2F).

Together, these analyses suggest that the TA life spans increase after GB, then shrink by about 2-folds for the following two divisions. Hence, contrary to the earlier assumptions, we find that germline transit-amplification is not a uniform process. Close to a previous estimate (Lindsley and and Tokuyasu, 1980), these methods indicated that the transit-amplification lasts for nearly 47 h (Table S6). A recent study in the *Drosophila* ovary also suggested anomalous alterations in the cell cycle structure during the TA stages, although the authors could not quantify the life spans (Hinnant et al., 2017). Similar developmentally regulated anomalous TA divisions were observed in the lineage of type II neuroblasts in *Drosophila*, in which the first daughter divides after 6.6 h and the matured late-stage daughters divide every 2–3 h (Homem et al., 2013). Together, these results suggest that the non-uniformity of the TA rates could be a recurrent theme in the TA lineages.

### Autonomous disruptions of G1/S or G2/M transitions in GSCs and GBs prolongs the life spans of all the TA stages

Next, to understand how changes in the life spans of stem cells and TA stages might influence the lineage progression, we estimated the life span changes because of autonomous perturbations of the cell cycle regulators in the GSCs and early TA cells. In *Drosophila* epithelial cells*,* Cyclin E promotes G1/S transition (Knoblich et al., 1994), and *String/CDC25* (*stg)*, via Cdk1 activation, induces the G2/M transition (Edgar and O’Farrell, 1989)*.* We showed earlier that RNAi knockdown of *cycE*, *stg*, and *cdk1* in the GSC and GB does not alter the transit-amplification program in testis (Gadre et al., 2020). Therefore, we probed the effects of these perturbations on the life spans of the GSC and TA stages.

**Figure 3:**
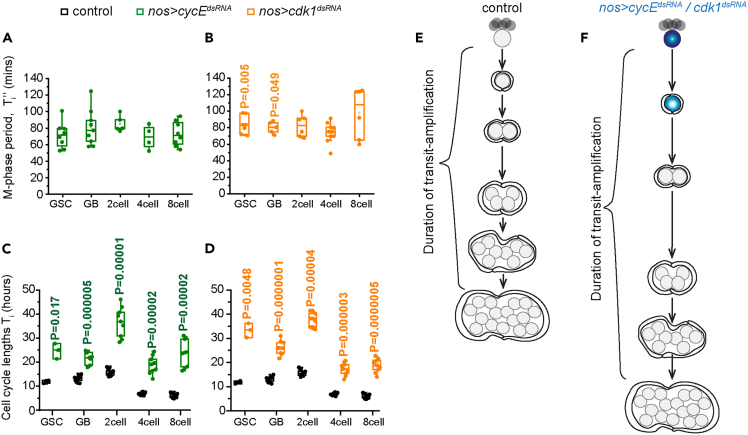
Autonomous disruptions of G1/S or G2/M transitions in GSCs and GBs prolongs the life spans of all the TA stages (A and B) Box plots show the duration of M-phase in GSCs and TA stages in *nos > hisRFP; Jup*^*PT*^ background overexpressing *cycE*^*dsRNA*^ (A; number of time lapse images analyzed = 27), and *cdk1*^*dsRNA*^ (B; number of time lapse images analyzed = 24). (p values calculated with respect to control in Figure 2 using Mann Whitney-U test). (C and D) Life span estimations in *nos > cycE*^*dsRNA*^ (C), and *nos > cdk1*^*dsRNA*^ (D) backgrounds (Mann Whitney-U test for 2cells in (D), otherwise Student’s t test). (E and F) Schematic summarizes the effects of the cycE and cdk1 RNAis in the GSCs and GBs on the TA progression. See also Figure S4.

We have shown earlier that *nosGal4vp16* driven overexpression of *cycE*^*dsRNA*^ perturbs CycE expression only in the GSCs and GBs (Gadre et al., 2020). In addition, time-lapse imaging suggested that only the M-phase durations of GSCs and GBs are significantly altered in the *nos > cdk1*^*dsRNA*^ background (Figure 3B). Therefore, we reasoned that the *nosGal4vp16*-mediated RNAi of CycE and Cdk1 affect their respective functions in the early stages. Hence, if the TA divisions are autonomously regulated independently of the GSC, the RNAi mediated knockdown of *cycE* or *cdk1* using *nosGal4vp16* should only increase the life spans of GSCs and GBs. Computation of the lifespans using the enumerations of the $\;D_{i+1}$’s (Table S3), the GSC mitotic indices (Table S1), and the $N_{i}$’s (Table S2 (Gadre et al., 2020)) in the *nos > cycE*^*dsRNA*^ and *nos > cdk1*^*dsRNA*^ backgrounds, respectively, revealed more than a 2-fold increase in the GSC and GB lifespans because of the *cycE* RNAi (*T*_*GSC*_ = 24.7 ± 3.2 h, *T*_*GB*_ = 21.5 ± 2.7 h; Figure 3C) and the *cdk1* RNAi (*T*_*GSC*_ = 33.3 ± 3 h, *T*_*GB*_ = 26.1 ± 2.9 h; Figure 3D). However, contrary to the expectation, both the RNAi perturbations also prolonged the lifespans of 2-cell, 4-cell, and 8-cell stages by a similar margin (Figures 3C and 3D), suggesting that the changes in the rates of the GSC/GB divisions are likely to influence those of the subsequent TA divisions (Figures 3E and 3F).

Similarly, though the *nos > stg*^*dsRNA*^ expression significantly reduced the intensity of anti-stg staining only in the GSCs (Figures S4A–S4C), it significantly reduced the GSC, GB and 2-cell population (Gadre et al., 2020). To understand how the loss of stg in GSCs could impact the rates of GSCs and subsequent TA stages, we estimated the M-phase durations in two different *nos > stg*^*dsRNA*^ backgrounds using the GFP-tub and Jup^PT^ reporters, respectively. Owing to synthetic lethality, we could only recover a single male fly of *UAS-GFP-tub; nos > stg*^*dsRNAI(III)*^ combination out of 118 expected class progenies (Figure S4F). The life span estimation using the data point predicted significantly higher cell cycle lengths of the GSC, GB, 4-cell and 8-cell stages (Figure S4H). Calculations based on the M-phase periods determined through the Jup^PT^ reporter with the *UAS-stg*^*dsRNA(II)*^ transgene inserted in the second chromosome and *nosGal4vp16* also yielded a similar set of predictions (Figures S4I–S4J).

Together, these results suggested that slowing down the cell cycle rates of GSCs and early TA stages could have a feedforward effect and prolong the subsequent divisions without altering the differentiation program after four rounds of TA.

### Ectopic Stg overexpression speeds up the entire transit-amplification program

**Figure 4:**
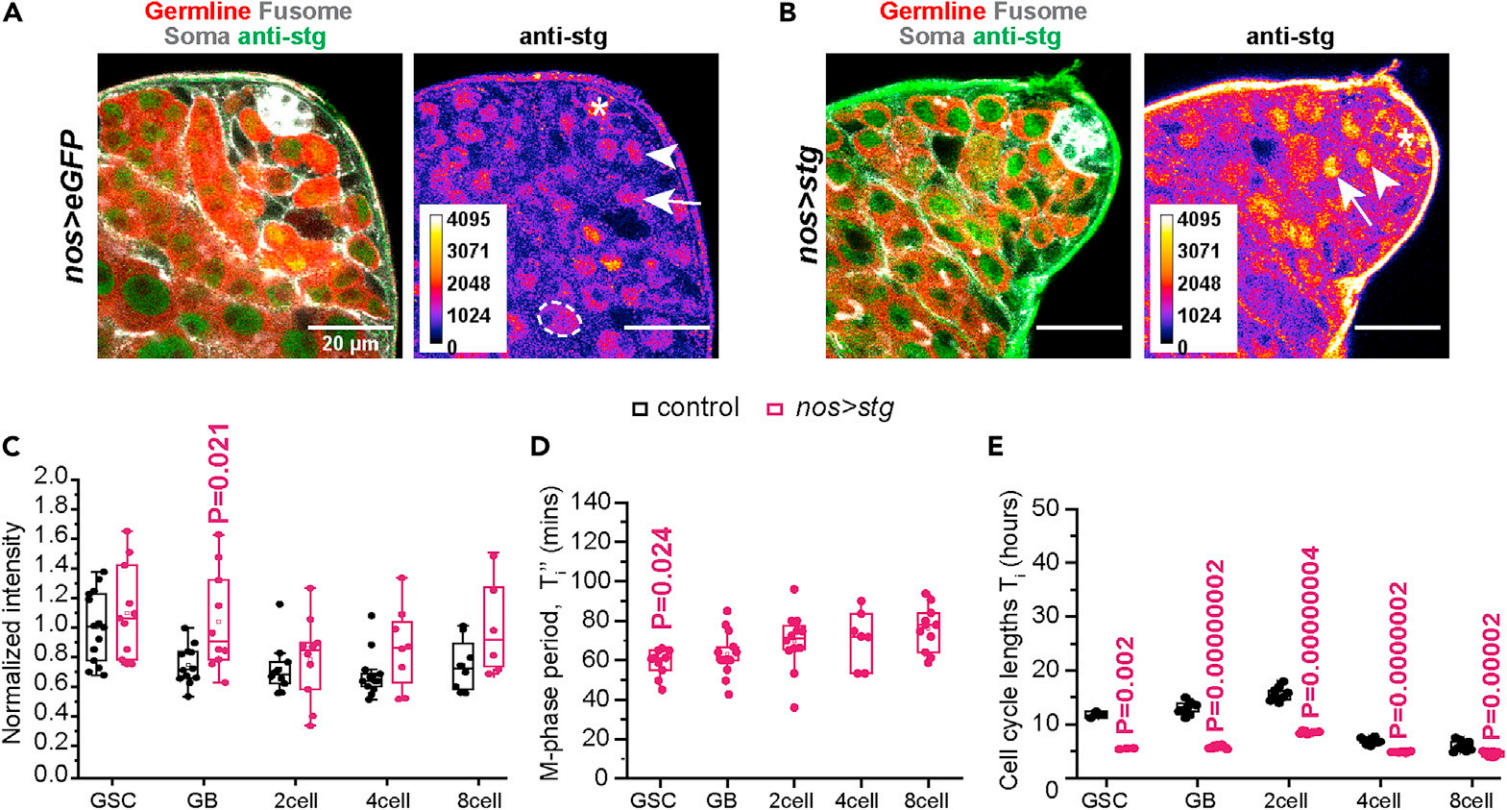
Autonomous acceleration of the G2/M transitions in GSCs and GBs shortens the life spans of all the TA stages (A and B) Single optical sections through the apical tip of adult testes from nos *> eGFP* (A) and *nos > stg* (B) backgrounds, respectively, depict the Vasa (red), hts1 (gray), armadillo (gray) and stg (green) immunostaining. Asterisk marks the hub, the arrowheads mark GSCs, the arrows mark GBs, and the dotted circle marks a spermatocyte. (Scale bars ∼20 μm). (C) The box plots indicate the stage-wise distribution of stg immunostaining intensities in the *nos > eGFP* (control) and *nos > stg* backgrounds (n ≥ 5; N ≥ 4). The n indicates the total number of cells analyzed, and N denotes the number of testes analyzed. The intensity of string immunostaining was normalized to the cellular area and divided by the staining intensity from a spermatocyte nucleus in the same z stack. (D) Box plots show the duration of M-phase in GSCs and TA stages in *nos > hisRFP; jup*^*PT*^ background overexpressing *stg*. (number of time lapse images analyzed = 25). (p values calculated with respect to control in Figure 2 using Mann Whitney-U test). (E). Life span estimations in control and *nos > stg* background (p values calculated using Student’s t test).

Ectopic Cdc25/stg overexpression at the 4- and 8-cell stages using *bamGal4* was shown to shorten the cell cycle lengths (Insco et al., 2009). The *nosGal4vp16* mediated *stg* overexpression significantly increased the intensity of stg-staining only in the GBs (Figures 4A–4C), shortened only the GSC M-phase period (Figure 4D), and significantly increased the GSC mitotic index (Table S1) (Gadre et al., 2020). Once again, these data suggested that the *nos > stg* overexpression could only perturb the Stg levels and its function in the GSC and GB stages. The life span estimation, however, indicated that the *stg* overexpression could not only shorten the lifespans of the GSCs (5.8 ± 0.2 h) and GBs (5.8 ± 0.3 h) by approximately 2-folds (Figure 4E), but it may also reduce the lifespans of 2-cell (8.5 ± 0.2 h), 4-cell (4.9 ± 0.1 h), and 8-cell (4.6 ± 0.5 h) stages by similar proportions (Figure 4E). Hence, we concluded that the shortening of the GSC and GB lifespans might influence the lifespans of later stages in the *nos > stg* background.

**Figure 5:**
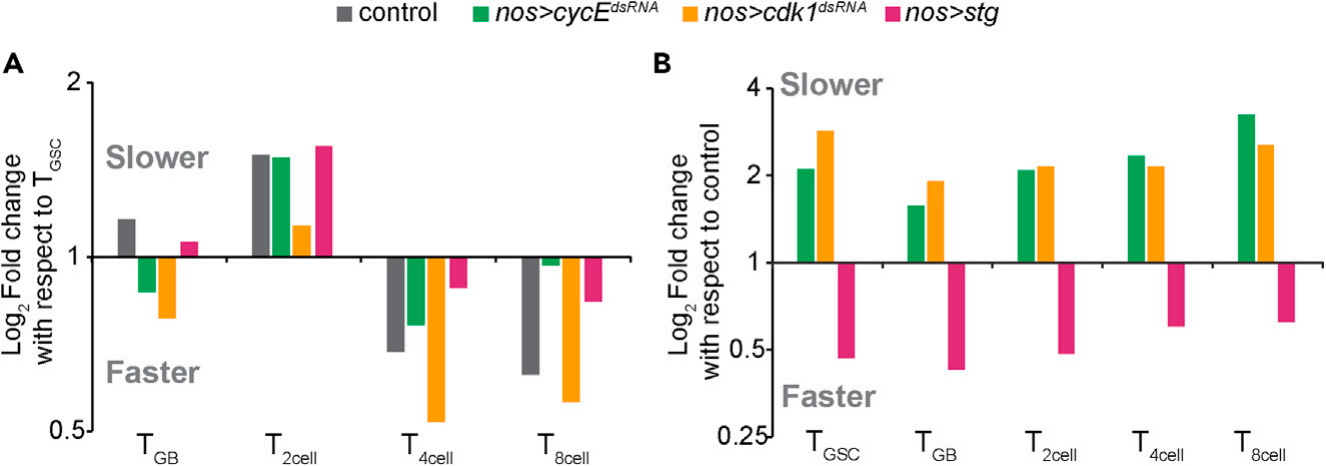
Fold-change in GSC and TA lifespans upon autonomous disruptions of G1/S or G2/M transitions (A) Log_2_ fold change in lifespans of TA stages in different genetic backgrounds relative to the GSC lifespans in respective backgrounds. Note that in control, the lifespans increase to about 1.5 fold and decrease by about 1.5 folds in 4- and 8-cell stages. (B) Log_2_ fold change in lifespans of GSCs and TA stages in different genetic backgrounds relative to control.

Together, these results suggested that perturbing the cell cycle lengths of GSCs and early TA stages would affect subsequent TA division rates, and consequently, the entire transit-amplification program (Table S6). We noted that alterations of the GSC life span because of the autonomous changes in the cell cycle regulators proportionately alter the lifespans of its lineage (Figure 5A), i.e., slower GSC divisions further slowed down the subsequent TA divisions (Figure 5B). Remarkably, more than 2-fold changes in the GSC lifespans and the overall TA period (Table S6) did not alter the lineage structure and the onset of meiosis after four cycles. Together, these observations further indicate that the transit-amplifying systems are highly resilient, and changes in the TA proliferation rates could regulate the onset of differentiation.

## Discussion

In the *Drosophila* testis, the GSCs can be easily identified because of the floral arrangement around the hub (Figures 1A and 1B), whereas the spermatogonial cysts are more abundant and tightly packed with no specific spatial marker for identification in live tissue. Hence, the time-lapse measurement of the cell cycle length was limited to the GSCs (Sheng and Matunis, 2011; Lenhart and DiNardo, 2015). The computation strategy described here estimates the average lifespans of the GSCs and TA cells at steady-state. This method requires time-lapse measurement of the M-phase period of GSCs and the persistence period of dead cysts. Because the M-phase in most adult cell types lasts for approximately 1 h (Georgi et al., 2002; Sigoillot et al., 2011; Padgett and Santos, 2020), this method considerably reduces the required duration of time-lapse imaging.

### The rates of germline cells anomalously slow down at the midpoint of transit-amplification

The persistence of c*ycE* expression was reported to alter significantly during the male germline transit-amplification in *Drosophila* (Gadre et al., 2020). This study also reported a significant increase in the mitotic index at the 4-cell stage. The life span predictions presented here match with the changes in the *cycE* expression and the mitotic index. Increased CycE persistence coincides with the life span extension at the 2-cell stage (Figure 5A), whereas reduced CycE persistence coincides with an increased mitotic index and life span reduction at the subsequent 4-cell stage (Figure 5A). This transition is marked by the termination of *held-out-wing* (HOW) (Monk et al., 2010) and the TFGβ signaling gradient (Kawase et al., 2004; Xia et al., 2010), as well as the onset of *bam* expression (Insco et al., 2009). Previous studies suggested that HOW maintains the Cyclin B levels in the *Drosophila* male germline (Monk et al., 2010), and Bam stabilizes Cyclin A in the *Drosophila* female germline (Ji et al., 2017). Loss of Bam or increased TFGβ signaling was suggested to slow down the male germline divisions at the 4-cell stage (Gadre et al., 2020). Together, these results indicate that the G1-S and G2-M transitions regulation would be crucial in controlling the cell cycle lengths at 2- and 4-cell stages.

Recent transcriptomic analysis of single-cysts in the *Drosophila* testis reported a relatively higher level of *wee1* (Cdk1 inhibitor) expression in the GBs and high levels of both c*yclinB3* and *twine (cdc25*) expression in the 4-cell cysts (Shi et al., 2020). Presumably, the *wee1* expression in GBs delays the G2/M transition, increasing the cell cycle length, whereas *cycB3 and twine* expressions in the 4-cell cysts would promote the G2/M transition, shortening the cell cycle length. What is the significance of these anomalous changes in cellular lifespans? Extended interphase at the 2-cell stage probably causes a cell-fate transition from a stem-cell mode to a TA mode. This extended interphase of the 2-cell cysts may facilitate the transcriptomic changes required for the impending induction of meiosis. Alternatively, the extension of the 2-cell interphase could be a consequence of the transcriptomic switch.

### Germline cells communicate with their daughters to regulate the rate of TA divisions

Previous studies proposed that the birth of the stem cell progeny sets a molecular clock that decides the differentiation time (Gao et al., 1997; Insco et al., 2009; Cinquin et al., 2010). This theory, however, failed to explain how a stereotypic differentiation clock fine-tunes along with the alterations in rates of TA divisions under different conditions (Lehrer et al., 1998; Di Gregorio et al., 2001; Charruyer et al., 2009; Paliouras et al., 2012; Giraddi et al., 2015; Ichijo et al., 2017; Tomasetti et al., 2019). Here, we show that 2 to 3-folds alteration in the lifespans of GSCs and GBs by autonomous perturbations of cell-cycle regulators also modifies the lifespans of the subsequent TA stages (Figure 5B) and resets the differentiation clock accordingly. As a consequence, the extent of germline amplification remains the same; thus, maintaining homeostasis. This conclusion, however, is at variance with the previous conjecture derived by altering the division rates of the late TA stages that induced a premature differentiation at the 8-cell stage or delayed the differentiation until the 32-cell stage (Insco et al., 2012; Gadre et al., 2020). One significant difference between the previous experiments and those described above is that we perturbed the stem cell division rate. The results imply that stem cells can send a forward signal to regulate the division rates of their TA daughters. A similar forward regulation has been reported in *Drosophila* intestinal lining (Ohlstein and Spradling, 2007) as well as mammalian tracheal epithelium (Pardo-Saganta et al., 2015). Together, these results suggest that the stem cells could potentially communicate with their daughters to regulate the differentiation program and homeostasis.

### Limitations of the study

We found significant fluctuations in the persistence time of a dead cyst of different TA stages (Table S4). A limited simulation showed that the variation in the persistence time (from 1.5 to 5 h) has no significant impact on the lifespan predictions (Figure S1C) because the average number of dead cysts in Phase-I was much smaller than total cysts in a given stage (Table S2). Hence, the method holds well in the case of a low frequency of cell death.

A previous study suggested that the GSCs undergo symmetric differentiation (13%_)_ and symmetric renewals (7%) with a low frequency (Sheng and Matunis, 2011). For this study, we have assumed that the frequencies of symmetric differentiation and symmetric renewals are negligible compared to the frequency of asymmetric division. However, we found that even a 10% change in the probability of asymmetric GSC divisions significantly alters the life spans predictions (Figure S5). Therefore, accurate measurement of the percentage of asymmetric GSC divisions will be essential to increase the confidence in the life span estimates.

Finally, the accuracy of this appraisal would depend on the sample size of each parameter and the penetrance of the genetic and environmental perturbations. Nevertheless, the method is internally consistent (Figures 2F; Tables S6 and S7), which would allow comparative and quantitative analysis to examine the effect of a perturbation.

## STAR★Methods

### Key resources table

**Table:** 

REAGENT or RESOURCE	SOURCE	IDENTIFIER
**Antibodies**		

Rat Monoclonal anti-vasa antibody	DSHB	Cat#anti-vasa, RRID:AB_760351
Mouse Monoclonal anti-armadillo antibody	DSHB	Cat# N2 7A1 ARMADILLO, RRID:AB_528089
Mouse monoclonal anti-Hts1/1b1 antibody	DSHB	DSHB Cat# 1b1, RRID:AB_528070
Rabbit polyclonal p-Histone H3(Ser-10) antibody	Santa Cruz Biotechnology	Cat# sc-8656-R, RRID:AB_653256
Guinea-pig anti-string antibody	Yukiko Yamashita (Inaba et al., 2011)	N/A
Goat Anti-Rat IgG(H+L) Cross-Adsorbed Secondary antibody, Alexa Fluor 488	Thermo Fisher Scientific	Cat# A-11006, RRID:AB_2534074
Goat Anti-Mouse IgG(H+L) Highly Cross-Adsorbed Secondary antibody, Alexa Fluor 568	Thermo Fisher Scientific	Cat# A-11031, RRID:AB_144696
Goat Anti-Rabbit IgG(H+L) Highly Cross-Adsorbed Secondary antibody, Alexa Fluor 647	Thermo Fisher Scientific	Cat# A-21245, RRID:AB_2535813

**Chemicals, peptides, and recombinant proteins**		

Hoechst 33342	Sigma	Cat#14533
Lysotracker RedDND-99	Life Technologies	Cat#L7528
Schneider’s insect medium	Sigma	Cat#S9895
Fetal Bovine Serum	GIBCO	Cat#16000-044
Penicillin/streptomycin	GIBCO	Cat#15140
Bovine insulin	Sigma	Cat#l0516

**Experimental models: Organisms/strains**		

*D. melanogaster*: CantonS	(Konopka and Benzer, 1971)	N/A
*D. melanogaster*: w[1118]; P{w[+mC]=GAL4::VP16-nos.UTR}CG6325[MVD1]	Bloomington Drosophila stock Center	BDSC: 4937 FlyBase: FBti0012410
*D. melanogaster*: w[1118]; P{w[+mC]=UAS-EGFP}34/TM3, Sb[1]	Bloomington Drosophila Stock Center	BDSC:5430 FlyBase: FBgn0014446
*D. melanogaster*: *UAS-cyclinE*^*dsRNA*^	VDRC; Austria	VDRC ID: 110204 (KK) FlyBase: FBgn0010382
*D. melanogaster*: *UAS-string*^*dsRNA(III)*^	VDRC; Austria	VDRC ID: 17760 (GD) FlyBase: FBgn0011354
*D. melanogaster*: *UAS-string*^*dsRNA(II)*^	VDRC; Austria	VDRC ID: 330033 (VSH) FlyBase ID: FBti0185702
*D. melanogaster*: *UAS-cdk1*^*dsRNA*^	VDRC; Austria	VDRC ID: 106130 (KK) FlyBase ID: FBgn0004106
*D. melanogaster*: UAS-string/ Cyo	Bloomington Drosophila Stock Center	BDSC:58439 FlyBase ID:: FBti0164791
*D. melanogaster*: Jupiter:GFP protein trap	(Karpova et al., 2006)	N/A
*D. melanogaster*: UAS-histone2A-RFP	Bloomington Drosophila Stock Center	BDSC:23650 FlyBase: FBti0077846
*D. melanogaster*: UAS-GFP-tubulin84B	Bloomington Drosophila Stock Center	BDSC:7374 FlyBase ID: FBgn0003884

**Software and algorithms**		

Fiji	Fiji	http://fiji.sc RRID:SCR_002285
GraphPad Prism 7.0	GraphPad Prism	http://www.graphpad.com/ RRID: SCR_002798
Microsoft Excel	Microscoft	https://www.microsoft.com/en-gb/ RRID: SCR_016137
Adobe Illustrator	Adobe	http://www.adobe.com/products/illustrator.html RRID: SCR_010279
Origin2020b	OriginLab	http://www.originlab.com/index.aspx?go=PRODUCTS/Origin RRID:SCR_014212

**Other**		

Raw datasets and confocal images	Mendeley Data	https://data.mendeley.com/datasets/nnmd48s389/draft?a=98a133af-5a16-4620-8b08-6b66be4c1d98 doi: https://doi.org/10.17632/nnmd48s389.2

### Resource availability

#### Lead contact

Further information and requests for resources and reagents should be directed to and will be fulfilled by the lead contact, Krishanu Ray (krishanu@tifr.res.in).

#### Materials availability

This study did not generate new unique reagents.

### Experimental model and subject details

#### Culture conditions

All stocks and crosses were maintained on standard *Drosophila* medium at 25°C. The method used for obtaining the vasa-positive TA stage-wise cyst count (from testes immunostained with anti-vasa and anti-armadillo) has been presented in our previous study (Gadre et al., 2020). The dataset used to compute GSC and TA stage-wise mitotic indices (sum of phosphoHistone-3 positive cysts divided by the sum of vasa-positive cysts at each stage) was presented in this study (Gadre et al., 2020). The list of fly stocks used in this study has been presented in the Key resources table.

### Method details

#### Whole-mount immunohistochemistry

Testes were dissected in Phosphate Buffer Saline (PBS) and fixed for 20-30 min with 4% paraformaldehyde. This was followed by the whole-mount immunohistochemistry protocol described previously from our lab (Joti et al., 2011). That is, the fixed samples were washed thrice with 0.3% Triton-X100 in PBS, immersed in blocking solution (5 mg/ml BSA in 0.3% Triton-X100 in PBS) for 30 min. The samples were then incubated with Rat monoclonal anti-Vasa (1:50 in Blocking solution, DSHB), Mouse monoclonal anti-Armadillo (1:100 in Blocking solution, DSHB), Mouse monoclonal anti-Hts1 (1:100 in Blocking Solution, DSHB) and Guinea pig anti-string (1:500 in Blocking solution) primary antibodies overnight at 4°C. The next day, the samples were washed 3 times in 0.3% Triton-X100 in PBS. The samples were then incubated for two-hours at room temperature with dye-conjugated Secondary antibodies (1:200 in Blocking solution, Thermo Fisher Scientific), followed by a final wash in in 0.3% Triton-X100 in PBS. The testes were mounted in a drop of Vectashield® (Vector Laboratory Inc., USA) between a glass slide and coverslip. Transparent nail paint was used to seal the edges of the coverslip.

#### Lysotracker staining

The Lysotracker RedDND-99 (Life Technologies) immunostaining was performed as previously described (Yacobi-Sharon et al., 2013; Yang and Yamashita, 2015; Chiang et al., 2017). That is, the dissected testes were incubated in Lysotracker RedDND-99 (Life Technologies) in PBS (1:1000) for 30 min on a shaker before 4% paraformaldehyde fixation.

#### *Ex vivo* imaging of testis

Testes from 4-day old flies were dissected and placed on a poly-lysine coated glass coverslip of a glass-bottom petri dish (P4707; Sigma Chemical Co. USA). For determination of the M-phase duration, the testes were immersed in Schneider’s insect medium (Sigma Chemical Co. USA) supplemented with 10% Fetal Bovine Serum (GIBCO, 16000-044), 0.5X penicillin/streptomycin (GIBCO, 15140) and 17 nM Bovine insulin (Sigma l0516) and imaged for 2 to 4 hours. The imaging interval was set as 5–6 minutes to minimize phototoxicity; therefore, the estimates have an intrinsic error of ± 5/6 minutes. To estimate the persistence time of dying cysts, dissected testes were incubated in Lysotracker RedDND-99 (ThermoFisher Scientific) in PBS (1:1000 dilution) for 30 minutes and then imaged in PBS for 3 to 4 hours. $A_{i}$ was re-defined as the phase-I to phase-II transition time, identified by the increase in Lysotracker staining intensity (Figure S1A) or the shrinkage of cell size (Figure S1B).

#### Image acquisition and analysis

Images were acquired using Olympus FV1000SPD laser scanning confocal microscope using 40X (1.3 NA), or Olympus FV3000SPD laser scanning confocal microscope using 60X (1.42 NA), 40X (1.3 NA), and 10X (0.4 NA) objectives. Multiple optical slices were collected to cover the entire apical part of the testis. Images were analyzed using ImageJ® (http://fiji.sc/Fiji). The Cell-counter^TM^ plugin was used for the quantification of the immunostained cysts.

### Quantification and statistical analysis

The sample sizes have been mentioned in the figure legends (Figures 1, 2, 3, 4, and S4) or tables (Tables S1–S3). To calculate the variation in the lifespan estimates, the medians of the first quartile and third quartile, and the overall median of the data for the M-phase period and vasa-positive counts were used. Student’s T-test was used to calculate P-values unless otherwise mentioned. Origin (OriginLab, Northampton, MA), Graphpad online software (https://www.graphpad.com/), and Microsoft Excel (2013) were used for statistical analyses.

## Data Availability

The TA stage-wise cyst counts in various genetic backgrounds used for lifespans estimations using Equation (7) have been presented in a previous study (Gadre et al., 2020). The entire dataset used for lifespans estimations using Equations (6) and (7), consisting of TA mitotic indices (Table S1), TA stage-wise cyst counts (Table S2), Phase-I germ-cell death (Table S3), persistence time (Table S4) and GSC and TA M-phase durations (Figures 2, 3, and 4; Table S5), has been presented in the main and supplement figures and supplemental tables. Original Excel files and image files used for the publication have been deposited at Mendeley and are publicly available as of the date of publication. The DOI is listed in the Key resources table. This paper does not report original code. The various equations used for the calculation of lifespans are presented in the main text (Equations (1)–(7)). A detail derivation of the equation is available on Mendeley data and DOI is listed in the Key resources table. Any additional information required to reanalyze the data reported in this paper is available from the lead contact upon request.

## Supplemental information

Document S1. Figures S1–S5 and Tables S1–S7

Video S6. ▪▪▪
