# Supplementary material for: The rates of stem cell division determine the cell cycle lengths of its lineage
Source: iScience. 2021 Oct 6;24(11):103232. doi: 10.1016/j.isci.2021.103232 (PMC8555441; doi:10.1016/j.isci.2021.103232)
Supplement: Document S1. Figures S1–S5 and Tables S1–S7 [file mmc1.pdf]

iScience, Volume *24*

Supplemental information

The rates of stem cell division determine the cell cycle lengths of its lineage

Purna Gadre, Nitin Nitsure, Debasmita Mazumdar, Samir Gupta, and Krishanu Ray

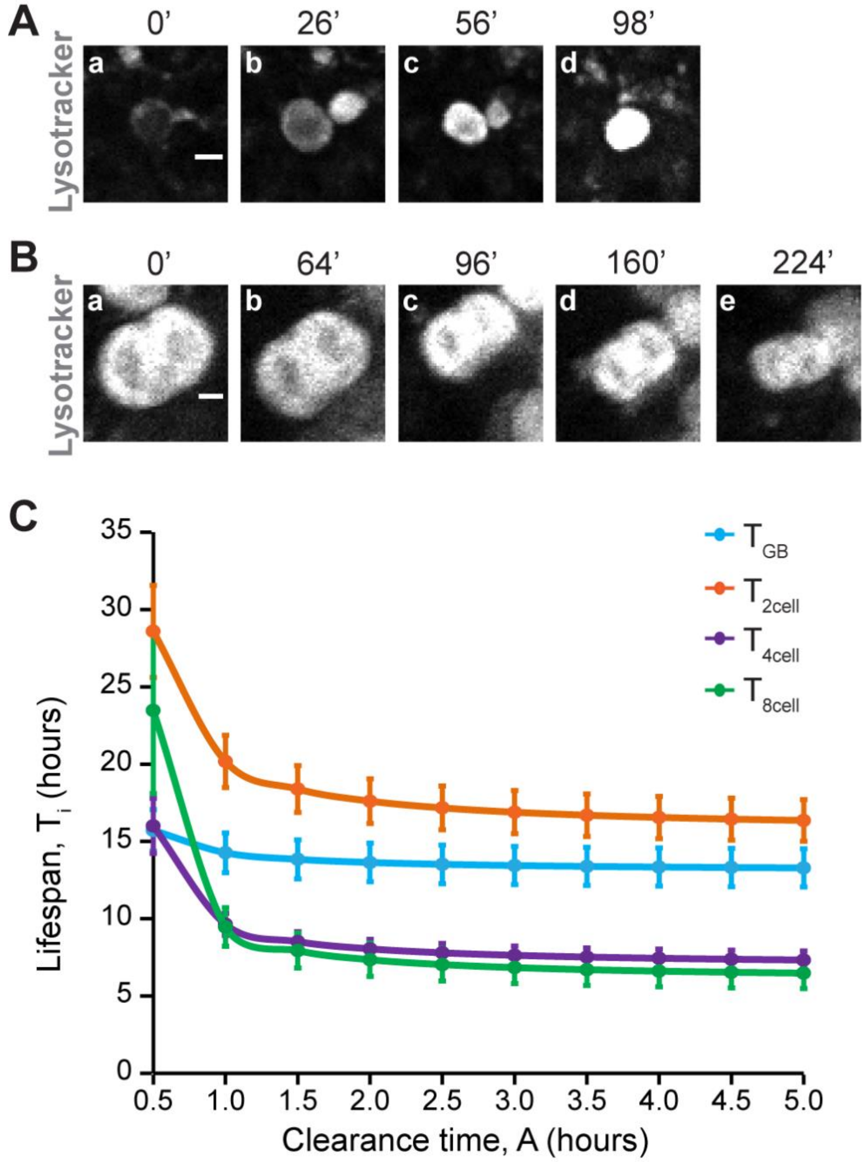

Figure S1. Persistence time of dead cyst (Related to Figure 2)

A) Montage of a GB transition from phase-I (a, b) to phase-II (c) of germ cell death, demonstrating an increase in the Lysotracker intensity. B) Montage of a 2-cell cyst transition from phase-I (a, b, c) to phase-II (e) of germ cell death, demonstrating size reduction. Time intervals in minutes are indicated at top of the panels. (Scale bar ~5**µ**m). C) Line plot shows predicted lifespans of GB ($\boldsymbol{T_{GB}}$, Blue), 2-cell ($\boldsymbol{T_{2cell}}$, Orange), 4-cell ($\boldsymbol{T_{4cell}}$, Purple), and 8-cell ($\boldsymbol{T_{8cell}}$, Green) in control (*nosGal4vp16>UAS-EGFP*) background with the persistence time ($\boldsymbol{A}$) ranging from 0.5 hours to 5 hours.

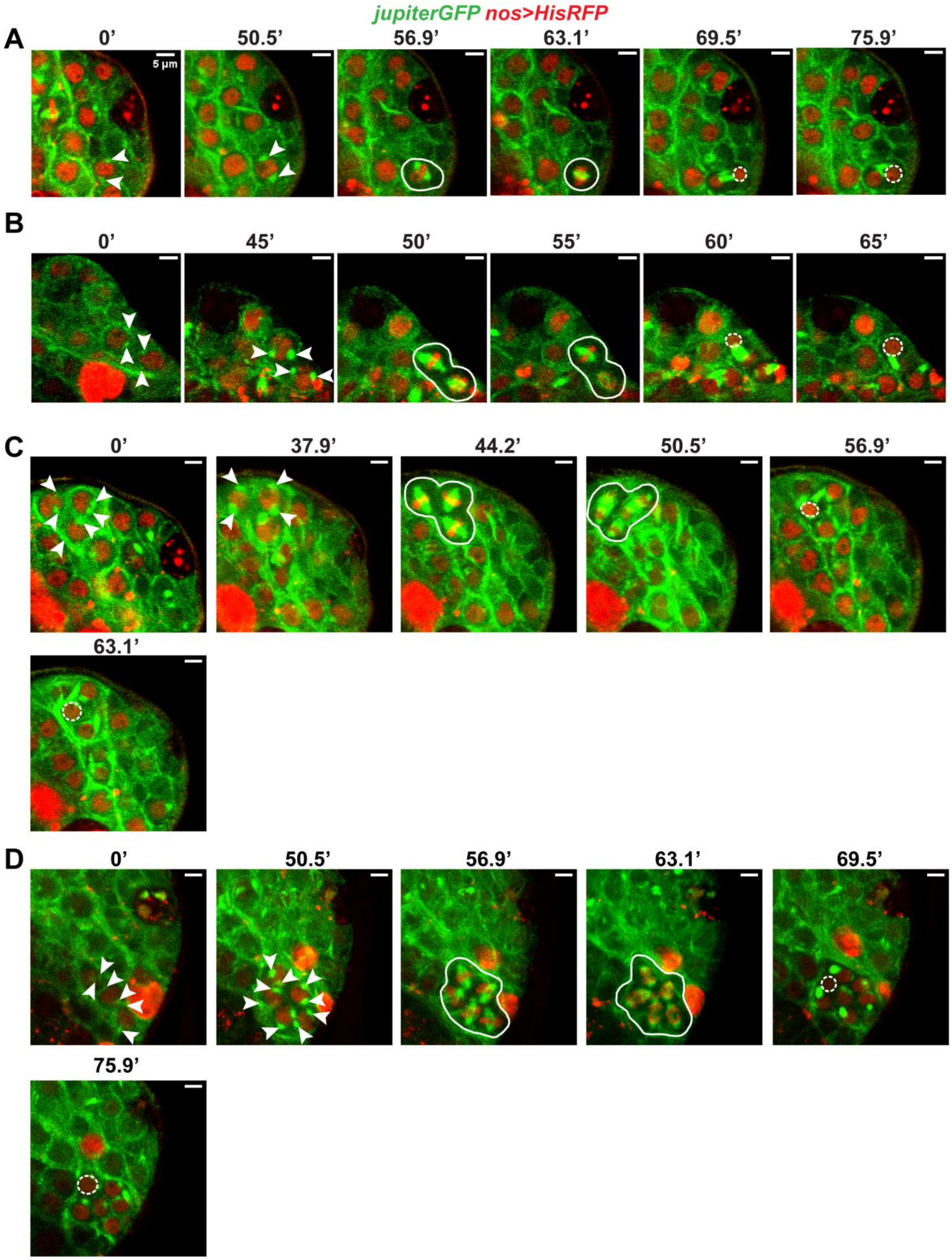

Figure S2. Time-lapse recording of M-phase in TA cells using Jupiter^{GFP} (Related to Figure 2)

Montage of a time-lapse image of a GB (A), 2-cell (B), 4-cell (C) and 8-cell (D) (*JupiterGFP* (Green), *nos>HisRFP* (Red)), undergoing M-phase. Arrowheads mark the position of the separated centrosomes. White circles label the cells visible in the plane of imaging. White dashed circles depict the increase in the nuclear size marking the end of telophase. Time intervals in minutes have been indicated on top of the panels. (Scale bars ~ 5µm).

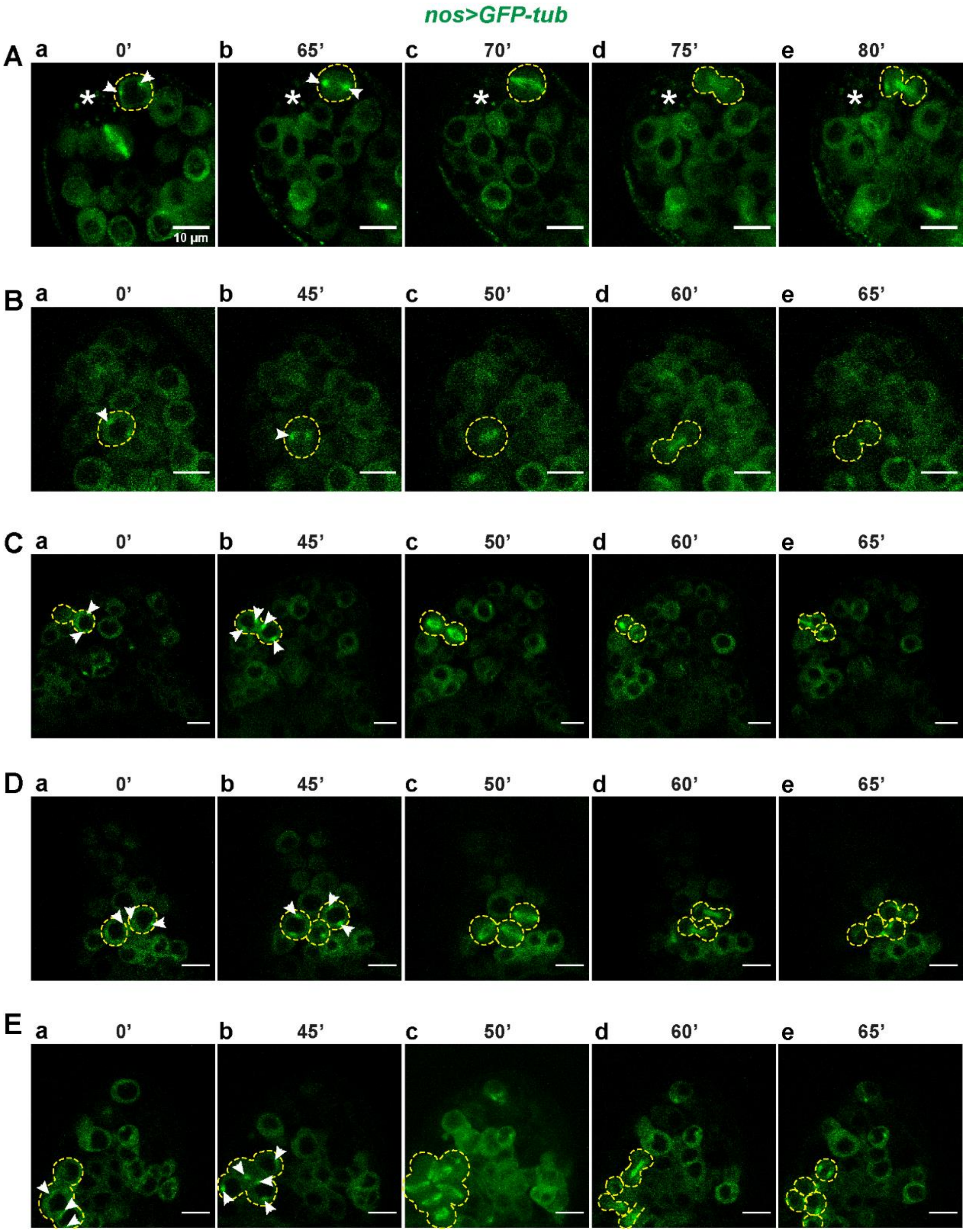

Figure S3. Time-lapse recording of M-phase in GSC and TA cells using GFP-tub (Related to Figure 2)

Montage of a time-lapse image of a GSC (A), GB (B), 2-cell (C), 4-cell (D) and 8-cell (E) (*nos>GFP-tub*(Green)) undergoing prophase (a), prometaphase (b), metaphase (c), and telophase (d, e). Arrowheads mark the position of the separated centrosomes. Yellow dashed circles show the position of the germline cell. Time intervals in minutes are indicated next to the image panels. (Scale bars ~ 10µm).

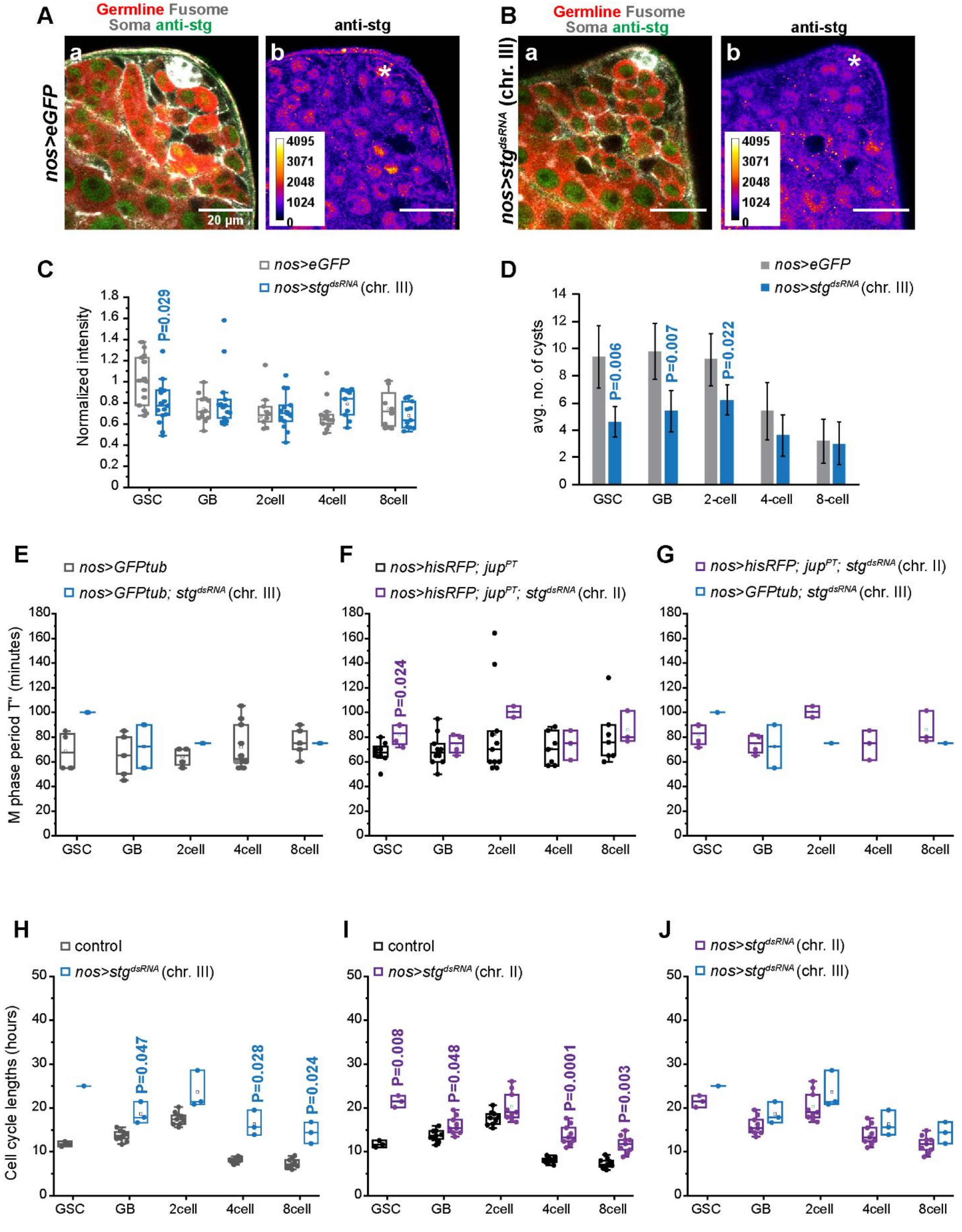
A
Germline Fusome
Soma anti-stg
anti-stg
nos>eGFP
20 µm
B
nos>stg^dsRNA (chr. III)
4095
3071
2048
1024
0
C
nos>eGFP
nos>stg^dsRNA (chr. III)
Normalized intensity
P=0.029
GSC GB 2cell 4cell 8cell
D
avg. no. of cysts
P=0.006
P=0.007
P=0.022
GSC GB 2-cell 4-cell 8-cell
E
nos>GFPtub
nos>GFPtub; stg^dsRNA (chr. III)
M phase period T'' (minutes)
F
nos>hisRFP; jup^PT
nos>hisRFP; jup^PT; stg^dsRNA (chr. II)
P=0.024
G
H
control
nos>stg^dsRNA (chr. III)
Cell cycle lengths (hours)
P=0.047
P=0.028
P=0.024
I
nos>stg^dsRNA (chr. II)
P=0.008
P=0.048
P=0.0001
P=0.003
J

23

Figure S4. Knockdown of *string* in GSCs prolongs the lifespans of all the TA stages (Related to Figure 3)

A-B) Apical tip of adult testis from nos>*EGFP* (A) and *nos>*$stg^{dsRNA(III)}$ (B) backgrounds stained with anti-Vasa (red), anti-hts1 (grey), anti-armadillo (grey) and anti-stg (green). Asterisk marks the hub. (Scale bars ~20µm). C) The box plots indicate stage-wise distribution of stg intensity in the *nos>EGFP* and *nos>*$stg^{dsRNA(III)}$ backgrounds (n≥9; N≥6). Where n equals the total number of cells analyzed and N equals the number of testes analyzed. The intensity of string staining was normalized to cellular area and divided by the area normalized string staining intensity of a randomly chosen spermatocyte from the same z-stack. D) The histogram shows the stage-wise average cyst distribution in *nos>EGFP* and *nos>*$stg^{dsRNA(III)}$ backgrounds. E) Box plots show the duration of M-phase in GSCs and TA stages in *nos>GFP-tub* and *nos>GFP-tub* (number of time lapse images analysed = 21), $stg^{dsRNA(III)}$ number of time lapse images analysed = 1) backgrounds. F) Box plots show the duration of M-phase in GSCs and TA stages in *nos>hisRFP;*jup^{PT} (number of time lapse images analysed = 26) and *nos> hisRFP;*jup^{PT}; $stg^{dsRNA(II)}$ number of time lapse images analysed = 18) backgrounds. G) Box plots show the duration of M-phase in GSCs and TA stages in *nos> hisRFP;*jup^{PT}; $stg^{dsRNA(II)}$ and *nos>GFP-tub,* $stg^{dsRNA(III)}$ backgrounds. H) Lifespans estimations in control and *nos>*$stg^{dsRNA(III)}$ backgrounds. I) Lifespans estimations in control and *nos>*$stg^{dsRNA(II)}$ background. J) Lifespans estimations in *nos>*$stg^{dsRNA(II)}$ and *nos>*$stg^{dsRNA(III)}$ backgrounds. (P values calculated using Students T-test).

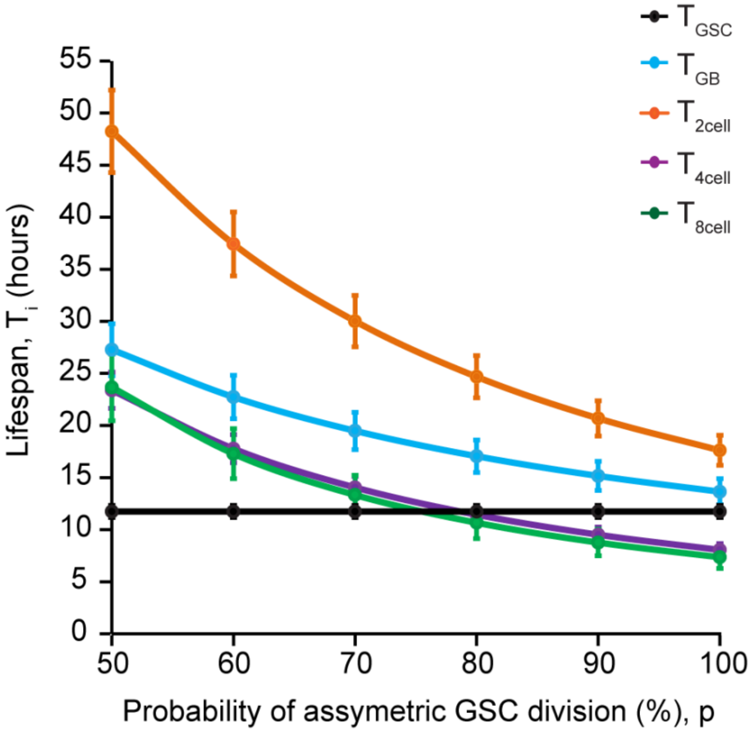

Figure S5. Variation in cellular lifespans with increasing probability of asymmetric GSC divisions (Related to Limitations of the study)

The line plot shows the predicted lifespans of GSC ($\boldsymbol{T_{GSC}}$, Black), GB ($\boldsymbol{T_{GB}}$, Blue), 2-cell ($\boldsymbol{T_{2cell}}$, Orange), 4-cell ($\boldsymbol{T_{4cell}}$, Purple), and 8-cell ($\boldsymbol{T_{8cell}}$, Green) in control (*nosGal4vp16>UAS-EGFP*) background with the % probability of asymmetric division ranging from 1 to 0.5 (q = 100, 90, 80, 70, 60, 50).

Supplement tables

Table S1: Stage-wise Mitotic index in control and cell cycle perturbed backgrounds (Related to Figure 2, 3)

Genotype	Mitotic index				
	**GSC**	**GB**	**2-cell**	**4-cell**	**8-cell**
nos> **control (n = 40)**	0.0962	0.0933	0.0639	0.1264	0.1373
nos>cycE^{dsRNA} **(n = 24)**	0.0482*	0.0629	0.0448	0.0870	0.0505
nos>Cdk1^{dsRNA} **(n = 21)**	0.0417[†]	0.0455	0.0500	0.0714	0.0909
nos>stg$^{dsRNA(III)}$ **(n = 23)**	0.0667	0.1074	0.0493	0.0787	0.1176
nos>stg **(n = 18)**	0.1835[‡]	0.2024[§]	0.1045	0.1707	0.1333

n = number of testes

* Fisher's exact test, *P* = 0.0476

† Fisher's exact test, *P* = 0.0312

‡ Fisher's exact test, *P* = 0.0116

§ Fisher's exact test, *P* = 0.0038

Table S2. Stage-wise cyst distribution profile (Median ± Interquartile range) (Related to Figure 2, 3)

Genotype	GSC	GB	2-cell	4-cell	8-cell
***nos>* control (n = 40)**	8 ± 1.25	9 ± 3	10 ± 3	5 ± 1.25	4 ± 2
***nos>cycE*^{dsRNA} (n = 25)**	9† ± 2	7† ± 2	9* ± 2	4 ± 2	4 ± 2
***nos>Cdk1*^{dsRNA} (n = 21)**	7 ± 2	6‡ ± 2	8† ± 2	4 ± 2	4 ± 2
***nos>stg*$^{dsRNA(III)}$ (n = 23)**	7† ± 1	5‡ ± 2	6‡ ± 3	4 ± 2	3* ± 1.5
***nos>stg* (n = 18)**	9* ± 2	9.5 ± 1	12† ± 2.75	7‡ ± 2	7‡ ± 3

n = number of testes

* Mann-Whitney-U test, $P < 0.05$

† Mann-Whitney-U test, $P < 0.01$

‡ Mann-Whitney-U test, $P < 0.001$

Table S3: Average number of cysts in Phase-I of Germ cell death (D_{i+1}) in different genetic backgrounds. (Related to Figure 2, 3)

Genotype	Average cell death (PHASE-I)				
	GSC	GB	2-cell	4-cell	8-cell
nos> **control (n = 54)**	0	0.06	0.09	0.04	0.06
nos>cycE^{dsRNA} **(n = 13)**	0	0.08	0.15	0	0.15
nos>Cdk1^{dsRNA} **(n = 29)**	0	0	0	0	0.03
nos>stg$^{dsRNA(III)}$ **(n = 21)**	0	0	0	0.05	0.1
nos>stg **(n = 10)**	0	0	0.4	0	0

n = number of testes

Table S4. Clearance time (A) recorded for different TA stages in various control genetic backgrounds. (Related to Figure 2)

Genotype	TA stage	Phase-I persistence time (Hours)
tj>	Gonialblast	>1.6
	Gonialblast	1.07*
	Gonialblast	>0.55
	Gonialblast	>0.95
	2-cell	>3.73
	2-cell	0.9*
	8-cell	>3.73
	8-cell	>1.27
	16-cell	>3.73
	16-cell	>2.20

The total number of time-lapse images acquired from the genetic controls were 71 - *tjGal4* (total 57) and *nosGal4* (total 14).

* Both the onset of Phase-I and transition to Phase-II were recorded

Table S5: Duration of M-phase, prophase, and metaphase-to-telophase (Median ± Interquartile Range) in different genetic backgrounds (Related to Figure 2, 3)

Genotype	GSC	GB	2-cell	4-cell	8-cell
	**M-phase duration (Hours)**				
nos>hisRFP;jup^{PT}	67.5 ± 7	67.5 ± 12	70 ± 24	70 ± 22	75.8 ± 21
nos>GFPtub	67.5 ± 26	65 ± 30	65.1 ± 11	65 ± 23	75 ± 15
nos>cycE^{dsRNA}***; hisRFP;jup***^{PT}	72.5 ± 18	78 ± 25	80.5 ± 11	70.3 ± 18	71.8 ± 23
nos>cdk1^{dsRNA}***; hisRFP;jup***^{PT}	83.7† ± 15	80.5* ± 11	82.6 ± 20	75.1 ± 11	108 ± 52
nos> stg$^{dsRNA(III)}$***; GFPtub***	100	72.5	75		75
nos>stg$^{dsRNA(II)}$***; his-RFP; jup***^{PT}	83.4* ± 14	75 ± 12	101 ± 5	75 ± 12	76.8 ± 13
nos>stg; hisRFP; jup^{PT}	61* ± 2	60 ± 12	71.2 ± 10	72 ± 9	77.9 ± 23

Genotype	Prophase duration (Hours)				
nos>hisRFP;jup^{PT}	50.3 ± 11	51.7 ± 15	50 ± 25	45 ± 24	56.9 ± 20
nos>GFPtub	52.5 ± 26	60.2 ± 15	47.6 ± 9	45 ± 15	55 ± 18
nos>cycE^{dsRNA}***; hisRFP;jup***^{PT}	53.3 ± 17	67.5 ± 28	68 ± 12	61.6 ± 22	56 ± 24
nos>cdk1^{dsRNA}***; hisRFP;jup***^{PT}	62.8* ± 18	59 ± 11	64.4 ± 24	60.9 ± 12	79.5 ± 47
nos> stg$^{dsRNA(III)}$***; GFPtub***	80	55	55		55
nos>stg$^{dsRNA(II)}$***; his-RFP; jup***^{PT}	55.6 ± 6	57.5 ± 19	74.7 ± 14	77 ± 41	56.3 ± 23
nos>stg; hisRFP; jup^{PT}	44.3 ± 15	48 ± 21	55.4 ± 15	56.3 ± 18	64 ± 16

Genotype	Metaphase-to-telophase duration (Hours)				
nos>hisRFP;jup^{PT}	15 ± 6	15 ± 5	15 ± 5	15 ± 6	15 ± 7
nos>GFPtub	15 ± 0	15 ± 0	20 ± 5	15 ± 1	15 ± 3
nos>cycE^{dsRNA}***; hisRFP;jup***^{PT}	15.9 ± 5	15.9 ± 8	15.4 ± 4	15.4 ± 5	16.1 ± 5
nos>cdk1^{dsRNA}***; hisRFP;jup***^{PT}	20.7* ± 4	20.8* ± 5	20 ± 6	16.1 ± 3	16.3 ± 5
nos> stg$^{dsRNA(III)}$***; GFPtub***	20	17.5	25		20
nos>stg$^{dsRNA(II)}$***; his-RFP; jup***^{PT}	20† ± 4	16.8 ± 12	16 ± 5	20 ± 3	20.5 ± 15
nos>stg; hisRFP; jup^{PT}	15.5 ± 5	15 ± 5	15 ± 5	15.5 ± 6	16 ± 5

* Mann Whitney-U test, $P < 0.05$
† Mann Whitney-U test, $P < 0.01$

Table S6: Lifespan estimations (Average ± SD) using the combination method in different genetic backgrounds (Presented in Figure 2, 3, 4 and 5)

Genotype	lifespan (Hours)					
	**GSC**	**GB**	**2-cell**	**4-cell**	**8-cell**	**Total TA**
nos>hisRFP;jup^{PT}	11.7 ± 0.6	13.6 ± 1.2	17.6 ± 1.4	8.1 ± 0.6	7.3 ± 1.1	46.6
nos>GFPtub	11.8 ± 2.3	13.7 ± 2.6	17.7 ± 3.5	8.1 ± 1.6	7.4 ± 1.8	46.9
nos>cycE^{dsRNA}	24.7* ± 3.2	21.5‡ ± 2.7	36.6‡ ± 6.2	18.8‡ ± 3.7	23.8‡ ± 5.6	100.7
nos>cdk1^{dsRNA}	33.3† ± 3	26.1‡ ± 2.9	37.7‡ ± 3	17.3‡ ± 2.6	18.7‡ ± 2.9	99.9
nos>stg$^{dsRNA(III)}$	25.0	18.7* ± 2.5	23.6 ± 4.3	16.3* ± 2.9	14.3* ± 2.5	72.9
nos>stg$^{dsRNA(II)}$	20.7† ± 1.8	15.4* ± 2.1	19.5 ± 3.4	13.3‡ ± 2.3	11.2‡ ± 2.0	59.4
nos>stg	5.5† ± 0.1	5.8‡ ± 0.3	8.5‡ ± 0.2	4.9‡ ± 0.1	4.6‡ ± 0.5	23.8

The Standard deviation was generated from variation observed in the TA stage-wise M-phase period (median, median of the 1st quartile and median of the 3rd quartile)

* Student's T test, $P < 0.05$

† Student's T test, $P < 0.01$

‡ Student's T test, $P < 0.001$

Table S7: Lifespan estimations (Average ± SD) using the equation (2) in different genetic backgrounds (Partially presented in Figure 2)

Genotype	lifespan (Hours)				
	GSC	GB	2-cell	4-cell	8-cell
nos>hisRFP;jup^{PT}	11.7 ± 0.6	12 ± 1.1	18.6 ± 3.1	9.2 ± 1.4	9.2 ± 1.3
nos>GFPtub	11.8 ± 2.3	11.6 ± 2.7	16.9 ± 1.5	9.1 ± 1.6	9.3 ± 0.9
nos>cycE^{dsRNA}	24.7* ± 3.2	20.6* ± 3.3	31.1† ± 2.2	14.3* ± 1.6	23.1† ± 3
nos>cdk1^{dsRNA}	33.3† ± 3	29.5‡ ± 2	27.2* ± 3.3	17.7† ± 1.3	18.5 ± 4.9
nos>stg$^{dsRNA(III)}$	20.9	11.2	25.4		10.6
nos>stg$^{dsRNA(II)}$	20.7† ± 1.8	11.6 ± 0.9	34† ± 0.8	15.8† ± 1.3	11.4 ± 1
nos>stg	5.5† ± 0.1	5† ± 0.5	11.3* ± 0.8	7.4 ± 0.6	9.3 ± 1.4

The Standard deviation was generated from variation observed in the TA stage-wise M-phase period (median, median of the 1st quartile and median of the 3rd quartile)

* Student’s T-test, *P* < 0.05

† Student’s T-test, *P* < 0.01

‡ Student’s T-test, *P* < 0.001
